# Manatee cognition and behavior: a neurobiological perspective on an unusual constellation of senses and a unique brain

**DOI:** 10.3389/fnbeh.2025.1576378

**Published:** 2025-04-11

**Authors:** Peter F. Cook, Gordon B. Bauer, Roger L. Reep

**Affiliations:** ^1^Marine Mammal Science, New College of Florida, Sarasota, FL, United States; ^2^Mote Marine Laboratory, Sarasota, FL, United States; ^3^Institute of Marine Sciences, Santa Cruz, CA, United States; ^4^Department of Physiological Sciences, University of Florida College of Veterinary Medicine, Gainesville, FL, United States

**Keywords:** manatee, cognition, neurobiology, senses, connectivity, brain, marine mammal

## Abstract

The nervous systems of manatees are strikingly unique across multiple dimensions. Manatees have the largest lissencephalic (smooth) brains in the animal kingdom, and demonstrate unusual somatosensory anatomy and physiology in the peripheral and central nervous system. As a rare aquatic herbivore sharing ancestry with modern elephants, manatee evolutionary history and behavioral ecology diverges substantially from that of other marine mammal clades, and their nervous system has adapted to the specific challenges they face. Although they are difficult to access for controlled behavioral study, prior neurobiological work has provided insight into manatee cognition. Here we review the evidence on manatee peripheral and central nervous function and present novel preliminary post-mortem diffusion MRI findings on whole-brain patterns of connectivity. Compared to another marine mammal, the California sea lion, manatee brains show apparently reduced corticocortical complexity. This may help explain their lissencephaly and relate to hypothesized reduced radial glial cell activity during neurodevelopment. Despite this apparently “simple” brain, manatees in the wild show some cognitively sophisticated behaviors, particularly in the realm of navigation. Future work in manatees should examine local and global brain connectivity related to spatial navigation and other complex cognitive capabilities.

## Introduction

Rapid speciation and adaptation to an aquatic environment has led to a range of novel neurobehavioral characteristics in the marine mammals (Cook and Berns, 2022; Cook et al., 2021; Herman, 2010; Kendall-Bar et al., 2023; Lyamin and Siegel, 2019; Oelschläger, 2008; Ridgway and Hanson, 2014). While substantial research has been conducted on the cognition^1^ of marine mammals (primarily cetaceans and pinnipeds), relatively little research has been conducted on manatees (Bauer and Reep, 2022; Henaut et al., 2022; Reep and Bauer, 2023), and that which has been done lies primarily in the area of sensory processes. This is despite the manifest uniqueness of manatee behavioral ecology and the manatee brain. Although we consider behavior to be the best indicator of cognitive capacity, the threatened, endangered or protected status of manatees throughout their range, and consequent policy protections, makes it difficult to conduct behavioral cognitive investigations in well-controlled laboratory environments. In contrast, neurobiological research that does not require live animals can be done more efficiently. Therefore, it is not surprising that much of the extant cognitive behavioral research with manatees was either preceded and motivated by reports of receptor neuroanatomy or expanded by synergistic neuroanatomy and behavioral research. Here, we summarize much of what is currently known regarding manatee neurobiology and contextualize it in relation to manatee behavior and cognition. We also put forth suggestions and preliminary results for further potentially fruitful avenues of neurobiological research with this understudied species.

Manatees belong to the order of the sirenians, herbivorous aquatic mammals which include two still living families: the trichechids and the dugongids (Nowak, 2003). The trichechids comprise three species of manatees (the Amazonian manatee, the West Indian manatee and the West African manatee), while the family of the dugongids includes the dugong and Steller’s sea cow (Nowak, 2003). Of these five species, only the first four still live, while Steller’s sea cow became extinct in the 18th century due to overhunting by humans (d’Isa and Abramson, 2025). Manatees and dugongs are unusual among marine mammals in being adapted to a lifestyle of aquatic herbivory. This has resulted in a collection of traits that is unique to sirenians, and that influences the form of their cognition and behavior (Reep and Bonde, 2021). These traits include large body size due to an expanded digestive tract, low metabolic rate, paddle-shaped fluke, absent hindlimbs, slow movement patterns, low encephalization quotient, lissencephaly, reduced visual and chemosensory systems, and expanded auditory and somatic sensory systems. The facial musculature is prominent and is used in feeding, which occupies several hours per day. Manatees exhibit exquisite spatial orientation in the wild, exemplified by several aspects of their navigational behavior. In the following manuscript, we briefly review extant data on manatee neurobiology, we consider how it likely bears on cognition and behavior, and we discuss potentially fruitful avenues for future research with this fascinating but understudied marine mammal.

### Sensory studies

The behavioral and neuroanatomical characteristics for sensory processes in manatees have been well-reviewed previously (Bauer and Reep, 2017; Marshall et al., 2022). In brief, the manatees are tactile and auditory specialists. Together with dugongs, they are the only mammals to have exclusively sensory hairs (vibrissae) covering their bodies (Reep and Bonde, 2021). Their exquisite hydrodynamic detection sensitivity, between a nanometer and micron particle displacement at low water movement frequencies directed rostrally (Gaspard et al., 2013) and an order of magnitude less sensitive postcranial, is mediated by these circumferentially receptive vibrissae (Gaspard et al., 2017). In the active tactile mode, they make fine discriminations of textures, operationalized as ridges and grooves, with their facial vibrissae, with a discrimination index (k) of 0.05 (Bachteler and Dehnhardt, 1999; Bauer et al., 2012), comparable to the sensitivity of the human index finger. These behavioral findings on the sensory character of manatee hairs were suggested earlier by Dosch (1915) and later by Reep et al. (2001, 2002) through careful analysis of the hair follicles before confirmation by behavioral testing. Sarko and Reep (2006) identified somatosensory mapping of the manatee body comparable to the human homunculus, which identifies another line for future behavioral investigation. B Although earlier evoked potential (e.g., Bullock et al., 1982) and anatomical analyses (Ketten et al., 1992) of manatee hearing suggested limited hearing abilities, subsequent behavioral studies indicated that similarly to other marine mammals, manatees have good hearing, including wide frequency sensitivity extending from 0.25 to 72 kHz (Gaspard et al., 2012; Gerstein et al., 1999), good directional hearing for broadband sounds, high temporal processing rates (600 Hz), and good ability to hear sounds in noise. Their temporal processing rate is lower than that of cetaceans, which unlike manatees echolocate, but considerably higher than terrestrial mammals (Mann et al., 2005). Chapla et al. (2007) identified the isolation of the ear from the skull, which explained the directional capabilities of manatee hearing.

Manatees are the only marine mammals with dichromatic color vision (Ahnelt and Kolb, 2000; Griebel and Schmid, 1996; Newman and Robinson, 2006). They have modest underwater visual acuity (Magnification Requirement, MAR: 20 arc minutes) (Bauer et al., 2003), a finding previously indicated by Mass et al. (1997) based on ganglion cell density and suggested by Reep and colleagues, based on modest development of visual pathways including the optic nerve, lateral geniculate nucleus, and cortical projection areas (Sarko and Reep, 2006; Sarko et al., 2007a). Limited visual acuity contrasts with good underwater resolution by pinnipeds (e.g., sea lions and harbor seals MAR: 4.7′–8.3′) (Schusterman and Balliet, 1970a; Barboza and Larkin, 2020b; Schusterman and Balliet, 1971) and cetaceans (bottlenose dolphin MAR: 8.2′) (Herman et al., 1975). The functional disparity in acuity between manatees and the other marine mammals is probably reduced by the enhancement of object resolution by color vision (Bauer and Reep, 2022). Interestingly, manatees probably share with other marine mammals the ability to see well both underwater and in air, although the mechanism for maintaining similar resolution probably differs.

Field observations suggest that manatees may make use of chemical senses in a variety of ways: identification of estrus females, localization of freshwater (through tracking saline gradients as do sea lions), and individual identification (reviewed in Bauer and Reep, 2022). Recent research on chemical receptors in the mouth supports the importance of taste sensitivity (Barboza and Larkin (2020b), although anatomical analysis of olfactory epithelium and neural pathways suggests only a modest sense of smell (Barboza and Larkin, 2020a). Formal psychophysical testing needs to be done to more accurately characterize the manatee chemical senses and compare them to the better studied dolphins and pinnipeds.

### Sensory hair system

Local cytoarchitectural organization can be examined practically and humanely in post-mortem brain tissue. Given the importance of somatosensation to manatee sensory ecology, somatosensory brain regions are of particular interest. Manatees possess only sensory hairs, about 2,000 on the face and head, and another 3,300 on the postcranial body (Reep et al., 1998). Each hair follicle is encapsulated by dense connective tissue, possesses a variety of mechanoreceptors that are densely innervated, and has a circumferential blood sinus like the vibrissae in other taxa (Reep et al., 2001, 2002; Sarko et al., 2007b). The facial hairs are larger and more densely innervated than the postfacial hairs, with an estimated total of ∼210,000 axons entering the CNS from the sensory hair follicles (∼110,000 on the face and head, and ∼100,000 axons from the follicles on the postcranial body) (Reep et al., 2001, 2002). As discussed above, the hairs on the face are used for direct contact tactile investigation, whereas both the postfacial and facial hairs are used for hydrodynamic detection of water movements. In addition, the largest hairs located in the corners of the mouth are used in conjunction with facial musculature to grasp food and bring it into the oral cavity, a process we call oripulation. Within the presumptive face representation in the cerebral cortex, large clusters of neurons are found in layer VI (Reep et al., 1989; Sarko and Reep, 2006). These were first described by Dexler (1913) in dugongs, and he named them Rindenkerne (“cortical nuclei”). Smaller Rindenkerne appear in presumptive non-facial regions of somatosensory cortex (Marshall and Reep, 1995). We have hypothesized that the Rindenkerne represent the sensory hairs, perhaps in a one-to-one fashion as is the case for the cortical barrels in layer IV of the face region of rodent somatosensory cortex, which represent the mystacial vibrissae. The extent of neural investment in processing information from the sensory hairs is also evident in the large sizes and extensive parcellation seen in somatosensory regions of the brainstem and thalamus (Sarko et al., 2007a). Because sirenians are considered to be somatosensory specialists, based upon observed behavior and anatomy, Rindenkerne may represent a variation that evolved independently of the barrels seen in other taxa that also use sensory hair-based somatosensation as a primary function.

### Navigation and spatial cognition

Additional aspects of cognition that have been well-studied in other marine mammals such as short-term memory, spatial memory, problem solving, sequence learning, symbolic communication, and imitation have not been formally studied in manatees, although we can make preliminary inferences from observed behaviors in the wild. For example, notable navigational capabilities associated with memory for warm water, freshwater, and food are exhibited by all three species of manatees [reviewed in Deutsch et al. (2022)]. They swim directionally over large expanses of open water (e.g., Big Bend area of Florida, United States) (Slone et al., 2022). They are also able to negotiate movement through the labyrinthine Ten Thousand Islands of Florida Bay (Haase et al., 2017; Stith et al., 2006). Travel through the maze-like waterways of the Amazon introduces a temporal and potentially cyclical aspect to manatee navigation, since they may have to remember routes and adjust for marked seasonal changes in water depth (Arraut et al., 2017). Deutsch et al. (2022) suggest that the failure of released manatees with no experience in the wild indicates that navigational knowledge is socially learned by calves from their mothers or conspecifics. However, spatial navigation involves multiple separate neurobehavioral systems, and may be supported by a range of neurobehavioral mechanisms with complex relationships to experience at different developmental time courses. Clearly more controlled behavioral data are needed to unravel the cognitive mechanisms of manatee navigation.

These abilities raise the possibility of sophisticated spatial navigation, including memory for frequently used complex trajectories. We suspect that the sensory hair system is involved in these behaviors, but this remains an untested hypothesis. If true, it would implicate somatic sensory regions of the brain, and higher order multimodal cortical areas in navigation. Some rodents use vibrissae in tactile exploration, and harbor seals and water rats have been shown to be able to track prey with the vibrissal sense. However, there are no data on vibrissal contribution to navigation in an open field environment. The relatively modest size of the hippocampal and parahippocampal areas of the manatee brain, both absolutely and relative to whole-brain size (Reep et al., 2007) raises the possibility that manatees may rely less on hippocampal spatial memory than some other species.

### Brain size

**FIGURE 1:**
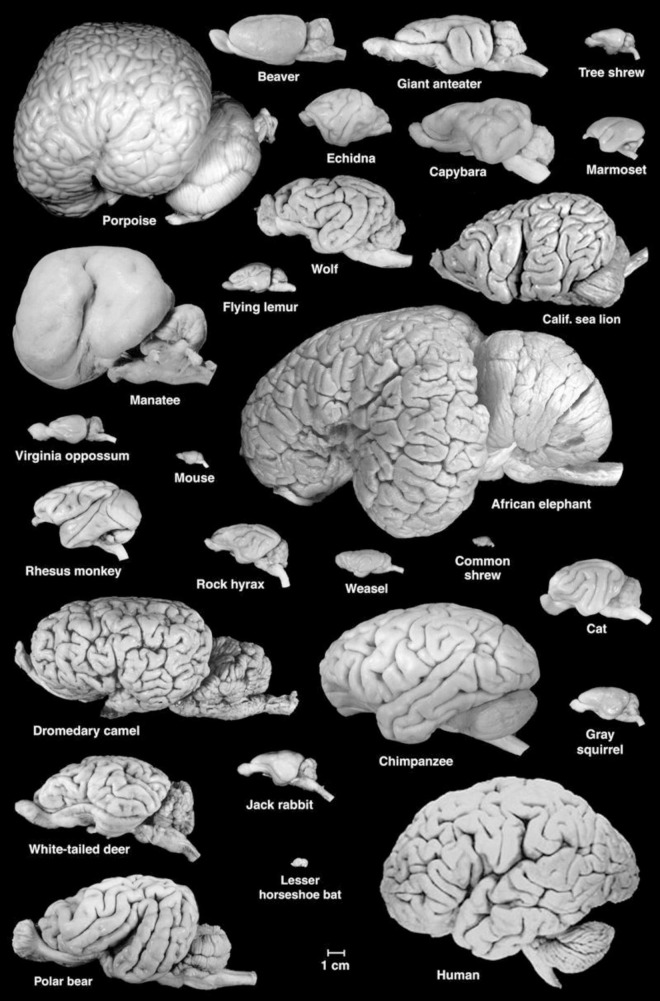
A collection of mammalian brains presented at the correct scale. Note the large size and lissencephalic condition of the manatee brain. Figure courtesy of Wally Welker and Carol Dizak.

Adult sirenian brains are about the size of a grapefruit, with those of Florida manatees weighing an average of ∼360 g (O’Shea and Reep, 1990). Thus, absolute brain size is large compared to that of many mammals (Figure 1). For many years, theorists argued that it was brain size relative to body size [or “encephalization quotient” (Jerison, 1973)] that best predicts cognitive capacity. Manatees have one of the lowest encephalization quotients among mammals, and it is particularly low when compared to some of the other marine mammals. O’Shea and Reep (1990) concluded that selection for large body size has resulted in low relative brain size in manatees. This is consistent with a lifestyle of aquatic herbivory, which requires an expanded digestive system. The resulting large body size is also advantageous for thermoregulation, which is critical given the low metabolic rate of manatees. Debate continues whether cognitive capacity is due more to small relative brain size or large relative body size (O’Shea and Reep, 1990). Regardless, newer analyses indicate that relative brain size is of limited value for broad interspecies comparisons (e.g., Deaner et al., 2007; van Schaik et al., 2021). Brain size is a crude measure at best, and other neural factors may be more relevant to understanding sensory and behavioral ecology and cognition.

### Lissencephaly in manatees

Within many mammalian lineages (e.g., carnivores) there is a trend toward increased gyration of the cerebral cortex with increased brain size (Pillay and Manger, 2007). The brains of species within a lineage often exhibit a pattern of gyri and sulci characteristic of that lineage, with smaller brains displaying fewer gyri and sulci and larger brains exhibiting more elaborate patterns superimposed upon the simpler one seen in smaller brains.

Strikingly, the cerebral hemispheres of sirenians are almost completely lissencephalic (smooth), with a gyration index of 1.06 (Reep and O’Shea, 1990). Many small mammalian brains are lissencephalic or nearly so, but manatees are extreme outliers, representing the largest brains above 100 g that are this lissencephalic (Mota and Herculano-Houzel, 2015). Lissencephaly is also seen in dugongs, and appears to have been a trait of sirenians throughout their evolution (Reep and O’Shea, 1990; Orihuela et al., 2019; Kerber and Moraes-Santos, 2021). The closest approximation to sirenian terrestrial ancestors are the now extinct, semi-aquatic quadruped sirenians *Prorastomus sirenoides* (Savage et al., 1994) and *Pezosiren portelli* (Domning, 2001). Each of these exhibits skulls suggestive of lissencephaly; there is no evidence of the groove-like undulations often seen in the skulls of gyrated brains. Earlier ancestors to the Sirenia are unknown. At the other end of the mammalian spectrum, cetacean brains are highly convoluted (gyration indices ∼5.0). It has long been known that cetacean cerebral cortex is thinner and contains fewer neuronal layers than most other mammalian brains. Because the cerebral cortex is thicker in manatees than in many other mammals (Reep and O’Shea, 1990), perhaps this trait drives the resulting lissencephalic condition. In an important study, Mota and Herculano-Houzel (2015) found that a single factor (the square root of cortical thickness multiplied by total cortical surface area) predicts the range of gyrification seen across all mammals including manatees. This brings manatees into the “fold” from their previous status as outliers, and suggests that factors that influence cortical thickness and surface area exhibit systematic variation that underlies the observed taxonomic differences in gyration. However, the question of why an animal with such a large brain has such high relative cortical thickness remains.

What is the significance of lissencephaly and related cortical thickness in manatees and dugongs? Is it the result of some peculiar pattern of neurogenesis or cellular migration in the developing cortex? Abnormal patterns of gyri and sulci, including lissencephaly, are seen in various human syndromes, are often associated with abnormal cortical cytoarchitecture, and have been linked to genetic and brain developmental abnormalities. However, cortical cytoarchitecture in adult manatees appears similar to that observed in many other mammals with large absolute brain size (e.g., carnivores and primates). Developmental studies in rhesus monkeys demonstrated that disruption of the normal pattern of gyration could result from altered cortico-cortical connectivity (Goldman-Rakic and Rakic, 1984). Could sirenian lissencephaly be related to the timing of the development of cortico-cortical connections? If this were the case, perhaps tension produced by cortico-cortical axon bundles (Van Essen, 1997, 2023) is applied too late to produce gyri in the thicker sirenian cortex.

Greater tangential migration of neuron progenitor cells increases cortical surface area and in gyrated brains neighboring cortical fields may exhibit different degrees of tangential migration. As has been demonstrated in ferrets, this localization of tangential expansion depends upon the density of basal radial glial cells (bRGCs) (Akula et al., 2023). The density of neuronal progenitor cells in the outer subventricular zone is predictive of the degree of cortical folding, and this density is directly related to the density of bRGCs. Furthermore, bRGCs promote tangential spreading by changing the architecture of the glial fiber scaffold. Finally, it has been shown that prolonging the proliferation of bRGCs leads to increased gyration, whereas reducing proliferation of bRGCs results in less gyration. This puts the focus on factors such as “Sonic Hedgehog” signaling that determine the cell cycle dynamics of bRGCs. Thus, if sirenian brains have a lower density of bRGCs in the developing cerebral cortex, they would not undergo as much tangential expansion. This could result in the greater cortical thickness seen in sirenian brains compared to gyrated brains.

The above paragraph applies to the primary gyri and sulci, which are the earliest to develop and are similar in geometry across individuals of a given species. Secondary gyri and sulci are more variable, occur later in development, and may be more influenced by individual-specific events including the distribution of thalamic and corticocortical connections.

The model of cortical expansion and folding promoted by Van Essen (2023) emphasizes the variety of factors that are likely to be involved. These include the mechanical properties of neural tissue (including cytoskeletal components), pressure exerted by cerebrospinal fluid in the lateral ventricles, and the tensional forces associated with axon bundles.

Our hypothesis is that manatees have reduced tangential expansion due to a paucity of bRGCs, producing a thicker cortex that is less prone to folding. Thus, primary gyri and sulci do not develop in the early phase of cortical development, and the tension produced by corticocortical connections is not sufficient to cause secondary gyri and sulci to form in the later phase of cortical development. Although this hypothesis is not realistically testable in manatees, the gathering of further related developmental data in other taxa will provide meaningful evidence. Regardless, due to emerging evidence about the role of RGCs in orchestrating whole brain connectivity (Casingal et al., 2022), reduced bRGC activity during neurodevelopment is likely to lead to unusual patterns of corticocortical organization.

### Brain connectivity

As discussed, most hypotheses bearing on the manatees’ lissencephaly involve factors related to neural migration and corticocortical connectivity patterns. While it is not feasible to examine these in manatees using chemical tracers or in vivo neuroimaging, advances in magnetic resonance imaging sequences allow high resolution diffusion tractography (Assaf and Pasternak, 2008) in fixed tissue (Miller et al., 2012). This allows ethically collecting brain connectivity data from opportunistically obtained, fixed samples. Diffusion tractography maps white matter connections in the brain and has been used successfully with other marine mammals, such as dolphins and whales, to examine patterns of cortical connectivity (e.g., Berns et al., 2015; Flem et al., 2024). Importantly, not only can opportunistic brain imaging help address existing neurobiological hypotheses, it can also directly support understanding of cognitive and behavioral processes. Prior cross-species evidence indicates conservation of core features of brain organization in related species with specific changes related to sensory ecology, sociality, and cognition (Goulas et al., 2019; Van Essen et al., 2019). Thus, mapping and comparing the brain organization of different species should give insight into similarities and differences in their behavioral ecology.

**FIGURE 2:**
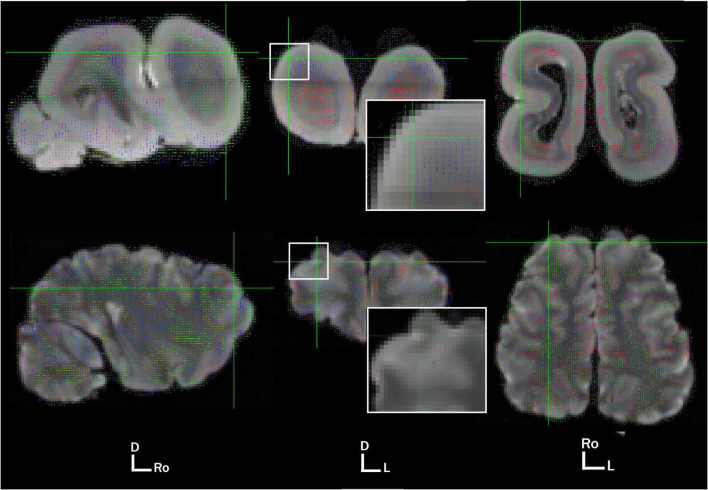
Primary voxel directionality at 1 mm in the West Indian Manatee **(top)** and California sea lion **(bottom)**. Views are sagittal, axial, and dorsal, moving from left to right. Brain orientation is represented by D, dorsal; Ro, rostral; L, left. Voxel directionality is represented by color and vector line: red, left to right; green, rostrocaudal; blue, dorsovental.

We present here preliminary diffusion data obtained opportunistically on a fixed, archival specimen (details on subject and imaging protocol in Supplementary materials). We imaged the brain at 3T using a specialized SSFP diffusion sequence as detailed in Berns et al. (2015). Diffusion data were obtained at 1 mm isotropic resolution. We compared the diffusion results to those previously acquired in a California sea lion (Cook et al., 2018). On first examination, one of the striking features of diffusivity in the manatee brain is the apparent concentric organization of directional bands of connection in the cortex and subcortex (Figure 2). It is unclear whether this is simply a byproduct of lissencephaly. However, despite their thick cortical sheet, manatees have less neuronal cell density in cortical layers than many species of comparable brain size (Charvet et al., 2016), which may allow larger aligned pyramidal axons to dominate the cortical diffusion signal. There is some evidence that, in other mammals, cortical regions show more homogeneous diffusion directionality earlier in development, prior to extensive synaptogenesis and myelination (Zhang et al., 2012).

**FIGURE 3:**
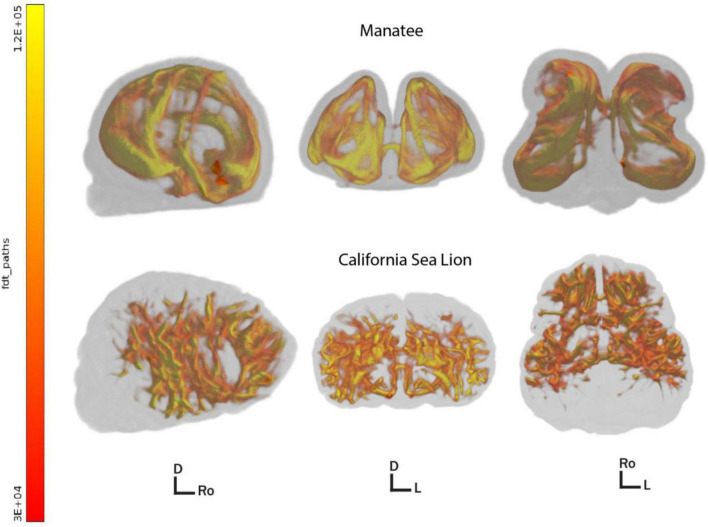
3D renders of thresholded whole brain corticocortical connectivity pathways in the manatee **(above)** and sea lion **(below)**. Brains are shown in the sagittal, axial, and dorsal orientation left to right. Directional indicators: D, dorsal; Ro, rostral; L, left. All paths above the minimum by-voxel streamline threshold of 3,000 are shown. Color represents regional streamline density, i.e., greater myelination and/or thicker axonal bundles. Images created with fsl’s render (Jenkinson et al., 2012).

In an effort to examine the patterns of corticocortical connectivity in the manatee brain, we also conducted whole-cortex tractography, using an algorithmic, anatomy-blind approach to segment cortex (not including cerebellum or sub-cortical gray matter) into 300 equally sized three-dimensional regions of interest. These were used to seed probabilistic tractography in fsl’s BEDPOSTX (Hernández et al., 2013). We used default settings to generate a whole-brain connectivity map and cortical region interconnectivity matrix mapping potential pathways between every region and every other. The same protocol was run on the manatee brain and a California sea lion brain. Whole-brain probabilistic tractography maps, thresholded at 3,000 streamlines, showed an apparently less complex distribution of interconnected cortical fibers in the manatee (Figure 3).

**FIGURE 4:**
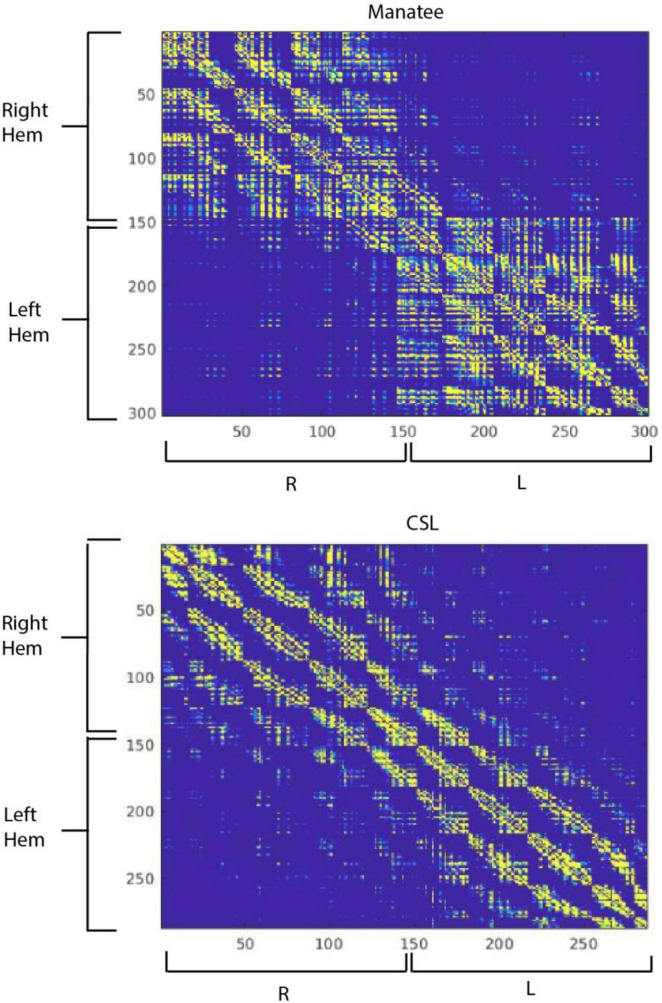
Corticocortical structural connectivity matrices in the manatee **(top)** and California sea lion **(bottom)**. Cortex was segmented algorithmically into 300 regions for each brain, numbered 1 through 300. The first region was right ventrofrontal, and the last left dorsocaudal. Regions were 9 × 9 × 9 mm in the manatee brain and 11 × 11 × 11 mm in the sea lion brain. The color at each point in the connectivity matrix represents in binary whether there was a connection between the corresponding points (regions) on the X and Y axis or not. Yellow represents a strong connection, blue a weak or no connection.

We then created a corticocortical connectivity matrix for both brains in MATLAB The MathWorks Inc (2022) (Figure 4). Connections between regions were thresholded at 1% of mean streamlines generated per region, and the matrices were then binarized for visualization. There were similarities and dissimilarities in the outputs. The manatee brain showed higher interhemispheric segregation than did the sea lion brain. Reduced interhemispheric connectivity in some marine mammals has been linked to unihemispheric sleep (Mascetti, 2016), but manatee sleep has not been studied. Within-hemisphere, the sea lion brain showed clearer segregation of a greater number of connectivity modules, indicating strong myelinated pathways connecting brain regions that are not immediately anatomically adjacent. The manatee’s apparent modules “bled together” more, suggesting more local connectivity and less organized global connectivity. We could not compute graph theory network complexity metrics on the manatee brain to compare to the sea lion brain, as the manatee brain graph was too sparse, or “disconnected.” This indicates that not all *a priori* regions were connected to the graph (i.e., sharing a myelinated pathway) at the binarization threshold we implemented. Importantly, this was a preliminary analysis, and brain regions were not functionally delineated. Also, the number of regions used in the analysis was arbitrary. Further, sparsely myelinated pathways are believed important to global connectivity and may not show up well in diffusion imaging (Santarnecchi et al., 2014).

Despite their preliminary nature, these findings paint a picture of a brain that may have less “complicated” (Sporns, 2007) global connectivity patterns. While this cannot, by itself, explain the manatee’s lissencephaly, it raises the intriguing possibility that the thicker cortical layer and reduced gyrification in the manatee are related to simplified connectivity patterns between disparate regions of the cortical sheet. Structural connectivity networks constrain emergent functional connectivity patterns associated with thought and action (Skudlarski et al., 2008; Ponsoda et al., 2016), and they vary over the course of development and evolution (Khundrakpam et al., 2013; Xu et al., 2020). As mentioned at the beginning of this section, the specific organization of the manatee brain will support the specific behavioral and cognitive needs of these species. Structural organization of the manatee brain might be compared to that of other marine mammals and terrestrial relatives (hyrax, elephant) to examine features of network organization bearing on cognition.

Reduced cortical complexity in the manatee could be due to reduced reliance on one or more sensory modalities and/or reduced need to integrate two or more sensory modalities. Hierarchical neural processing also relies on long-distance white matter pathways (Harris et al., 2019), connecting regions at the top of the motor hierarchy (e.g., prefrontal cortex) and regions at the top of the sensory hierarchy (e.g., hippocampus) with polymodal and unimodal sensory and motor regions. Sparser connectivity between these regions might indicate a brain less reliant on top-down processing. It bears emphasis that, in the limited studies completed, manatees show good capability for learning (Bauer and Reep, 2022) and have excellent hearing and somatosensory capability. However, they have modest visual acuity (Bauer et al., 2003; Mass et al., 1997) and apparently simple foraging and social ecology (Henaut et al., 2022). Conditioned learning is largely dependent on subcortical structures and subcortical-cortical connections, and likely does not depend heavily on corticocortical connectivity patterns (Grossberg, 2009). Behavioral and cognitive studies of manatee sensory integration, experiential memory, and cognitive control would provide data that might covary more tightly with specific characteristics of manatee cortical organization and intracortical connectivity. Sea lions, the species to which we compared the manatee’s corticortical connectivity, have distinguished themselves in terms of performance on laboratory tests of cognition (Cook et al., 2021), and appear to have much more complex social and foraging ecology than do manatees. Finally, although is has been difficult to definitively identify and validate behavioral operationalization of complex cognitive phenomena in non-human animals, specific cognitive domains are likely linked with specific brain regions and circuits (Petersen and Sporns, 2015), and these could be examined together. For example, manatee hippocampal connectivity and microcircuitry may underlie their capability for flexible deployment of experiential and spatial memory during navigation. Navigational experiences may in turn modulate hippocampal connectivity, potentially increasing it. Careful behavioral studies of spatial memory and hippocampal structure and organization may inform each other, helping determine to what extent manatees are relying on typical mammalian hippocampal supported spatial memory in finding their way around.

## Conclusion

A truly “comparative” neuroscience must extend well beyond typical laboratory rodent and fish models. Due to rapid speciation, the demands of adapting a mammalian bauplan to an aquatic or semi-aquatic lifestyle, and the broad range of gross neural adaptations and extremes found in marine mammals, they represent a highly promising set of clades to uncover and illustrate the effects and limits of adaptive pressure working on nervous systems (Bauer et al., 2020). Among the marine mammals, manatees are particularly understudied. As we have suggested here, manatee brains are strikingly unusual, at both the cellular and whole brain network level. Future work should continue to attempt to link field and laboratory observations of behavior with specific neural adaptations. Promising avenues to explore include sensory processing, spatial navigation, and flexible cognition/executive function. Future connectivity studies could inform behavioral and cognitive hypotheses. The cortical targets of the hippocampus and MTL ought to depend on the types of sensory representations that populate manatee spatial and event memory. Looking at the connectivity of somatosensory cortex could inform hypotheses about sensory integration. Manatees will continue to be difficult species with which to conduct laboratory and field research into cognition. As a field, comparative neuroscientists should make better use of neurobiological data that is increasingly available.

## Data Availability

The original contributions presented in this study are included in this article/Supplementary material, further inquiries can be directed to the corresponding authors.

## Footnotes

1 Cognition broadly considered includes attention, perception, learning, memory, problem solving, and decision making (Shettleworth, 2009).
