# Supplementary material for: Manatee cognition and behavior: a neurobiological perspective on an unusual constellation of senses and a unique brain
Source: Front Behav Neurosci. 2025 Apr 11;19:1576378. doi: 10.3389/fnbeh.2025.1576378 (PMC12021893; doi:10.3389/fnbeh.2025.1576378)
Supplement: Supplementary file 1 [file Data_Sheet_1.pdf]

Supplementary Methods

Samples

Both brains imaged in this study were obtained opportunistically. Manatee MJAV9034 was a wild adult male who died in July of 1990, due to net entanglement. He was 486 kg, 295 cm in length. The brain was fixed in buffered formalin before being sent to Emory University in 2016. California sea lion CSL 10814 was an adult female who stranded in central California in early 2014. She was sent to The Marine Mammal Center in Sausalito, CA where she was diagnosed and treated for urogenital carcinoma before euthanasia on March 14, 2016, for medical cause. The brain was removed by a veterinary pathologist, cold fixed in 10% buffered formalin for a month, then shipped to Emory University.

Imaging

Sequences

The post-mortem imaging method is detailed in Berns et al., 2015. In brief, brains were imaged post-mortem in agarose doped with gadolinium. They were imaged in a 32-channel head receive coil on a 3 T Siemens Trio scanner with standard gradients. We used a diffusion-weighted, steady-state free precession (DW-SSFP) sequence optimized for post-mortem tissue (Miller et al., 2012). Fifty-two diffusion directions were collected for each brain, with the following parameters: FOV: 128 mm, voxel size: 1 mm isotropic, TR: 31 ms, TE: 24 ms, flip angle: 35 degrees, bandwidth: 159 Hz/pixel, q: 255 cm^{-1}, G_{max}: 38.0 mT/m, gradient duration: 15.76 ms. Effective b = 0 images were also acquired for each brain with a q = 10 cm^{-1} scan applied in one direction only. DW-SSFP diffusion modeling requires calculating T1 and T2 values, which were calculated with T1-weighted and T2-weighted images. TIR sequences were obtained with TR: 1,000 ms, TE: 12 ms, and TI: 30, 120, and 900 ms. TSE sequences were obtained with TR: 1,000 ms, TE: 14, 29, and 43 ms. All diffusion pre- and post-processing, was conducted with modified FSL tools (Jenkinson et al., 2012) accounting for the DW-SSFP model and incorporating T1 and T2 measures.

Berns, G. S., Cook, P. F., Foxley, S., Jbabdi, S., Miller, K. L., & Marino, L. (2015). Diffusion tensor imaging of dolphin brains reveals direct auditory pathway to temporal lobe. *Proceedings. Biological Sciences*, *282*(1811), 20151203. https://doi.org/10.1098/rspb.2015.1203

Jenkinson, M., Beckmann, C. F., Behrens, T. E., Woolrich, M. W., & Smith, S. M. (2012). Fsl. *Neuroimage*, *62*(2), 782-790.

Miller, K. L., McNab, J. A., Jbabdi, S., & Douaud, G. (2012). Diffusion tractography of post-mortem human brains: Optimization and comparison of spin echo and steady-state free precession techniques. *NeuroImage*, *59*(3), 2284–2297. https://doi.org/10.1016/j.neuroimage.2011.09.054
